# Time spent on work-related activities, social activities and time pressure as intermediary determinants of health disparities among elderly women and men in 5 European countries: a structural equation model

**DOI:** 10.1186/s12939-018-0840-y

**Published:** 2018-08-16

**Authors:** Nicholas Kofi Adjei, Kenisha Russell Jonsson, Tilman Brand

**Affiliations:** 1 Department of Prevention and Evaluation, Leibniz Institute for Prevention Research and Epidemiology – BIPS, Unit Social Epidemiology, Achterstrasse 30, D-28359 Bremen, Germany; 2 Health Sciences Bremen, University of Bremen, Bremen, Germany; 3 Department of Sociology, University Essex, Colchester, UK; 4 Department of Health Sciences, Africa University, Mutare, Zimbabwe

**Keywords:** Psychosocial factors, Self-reported health, Gender, Elderly, Time-use activities, Stress

## Abstract

**Background:**

Psychosocial factors shape the health of older adults through complex inter-relating pathways. Besides socioeconomic factors, time use activities may explain gender inequality in self-reported health. This study investigated the role of work-related and social time use activities as determinants of health in old age. Specifically, we analysed whether the impact of stress in terms of time pressure on health mediated the relationship between work-related time use activities (i.e. housework and paid work) on self-reported health.

**Methods:**

We applied structural equation models and a maximum-likelihood function to estimate the direct and indirect effects of psychosocial factors on health using pooled data from the Multinational Time Use Study on 11,168 men and 14,295 women aged 65+ from Italy, Spain, UK, France and the Netherlands.

**Results:**

The fit indices for the conceptual model indicated an acceptable fit for both men and women. The results showed that socioeconomic status (SES), demographic factors, stress and work-related time use activities after retirement had a significant direct influence on self-reported health among the elderly, but the magnitude of the effects varied by gender. Social activities had a positive impact on self-reported health but had no significant impact on stress among older men and women. The indirect standardized effects of work-related activities on self-reported health was statistically significant for housework (*β* = − 0.006; *P* < 0.001 among men and *β* = − 0.008; *P* < 0.001 among women) and paid work (*β* = 0.012; *P* < 0.01 among men and *β* = 0.000; *P* > 0.05 among women), which implied that the paths from paid work and housework on self-reported health via stress (mediator) was very weak because their indirect effects were close to zero.

**Conclusions:**

Our findings suggest that although stress in terms of time pressure has a direct negative effect on health, it does not indirectly influence the positive effects of work-related time use activities on self-reported health among elderly men and women. The results support the time availability hypothesis that the elderly may not have the same time pressure as younger adults after retirement.

**Electronic supplementary material:**

The online version of this article (10.1186/s12939-018-0840-y) contains supplementary material, which is available to authorized users.

## Background

Gender differences in health among the elderly have been reported in several studies [1–4]. Similar findings among younger adults [5, 6], affirms the long-standing health-survival paradox that women live longer than men, yet they report poorer health [7, 8]. However, there is some evidence that suggests non-existence of gender differences in self-reported health among the elderly in some high-income countries such as the UK, the US and Finland [9, 10]. Thus, the paradox may be country-specific as previously shown for some welfare countries [11]. Furthermore, health differences may be dependent on the health outcomes and phase of the life cycle [9]. The reasons for these observed gender disparities in health are complex and interrelated, but the most cited explanations are differences in biological traits [12–14]. Nonetheless, epidemiological research suggests that biological factors are not sufficient in explaining the health gap between women and men [12, 15].

From a health inequality perspective, several explanatory factors have been suggested [3, 15–17], most of which have been linked to differences in socioeconomic positions such as education, income, and occupation as the main sources of inequality between men and women. Studies have shown that socioeconomic position is often lower among women and thus they are exposed to high levels of stress [18], and among the elderly, they are exposed to a wide range of psychosocial risk factors, when in a lower socioeconomic position [19]. Furthermore, the differential vulnerability hypothesis also suggests that there may be variations by gender in vulnerability to behavioral and psychological health conditions [2].

Notwithstanding the importance of biological and socioeconomic forces in explaining the health disparities between men and women, these factors may not be sufficient for understanding the health gap seen between elderly persons of different gender. A further explanation may be linked to results from post-retirement time use studies which revealed that older men and women often are engaged in social roles and activities such as housework activities [20, 21], leisure activities [20, 22] and voluntary work [23–25] to different degree.

In this regard, social roles and the time invested in such activities, summarized here as time use activities, may to some extent explain the gender differences in health [26]. Studies that used the concept of social roles such as marital status (i.e., being married, divorced, separated or widowed), to examine these relationships concluded that social roles that people occupy may have an impact on their health [27, 28]. However, Bird and Fremont [26] have pointed out that these indicators of social roles are crude and indirect and thus time and effort spent on social roles and activities should be investigated.

Among the elderly, time use activities may be an important determinant of health considering the time availability after retirement [20, 29]. Interestingly, evidence suggests that gender inequality in work-related time use activities (i.e., paid work and housework) persist in high-income countries even after retirement [11, 30]. While elderly men allocate more time to paid work, older women allocate more time to housework activities [29, 31, 32], even though time allocated to housework activities among older men has increased over the years [31]. Regarding these household activities, men typically perform the occasional tasks while women are responsible for routine housework [33, 34]. Despite the gender differences in the distribution of housework, performing these activities are deemed “productive activities” [35] because they are activities that older adults might have delegated to a paid worker.

The inequitable distribution of work-related time use activities may be a contributing factor for the observed gender health differences [36, 37]. Although moderate time spent on these activities can be beneficial to health among the elderly [11], Luoh and Herzog [38] suggested that longer hours devoted to paid work activities might not necessarily improve the health among the elderly. In a recent study Adjei and Brand [39] concluded that older women have higher odds of reporting poor health when more time is devoted housework combined with either short or long sleep duration. The combination of more hours of housework and paid work activities has also been shown to be more stressful among women [40]. Moreover, longer time allocated to these activities may increase time pressures [41]. It may also reduce time availability for social activities such as participation in clubs and religious involvement [42], which may have positive health effects.

From the above discussions, it is clear that the literature on socioeconomic status, work-related activities and stress have identified a direct relationship with health among older adults. However, we argue that these psychosocial factors may have an indirect differential impact on health among men and women. Furthermore, stress in terms of time pressure can mediate the associations between health status and work-related activities, but we speculated in our previous papers [11, 39] that the strength of the relationship between these activities and self-reported health via stress might be weak, due to time availability at old age. However, this assertion has not yet been supported with empirical data among elderly men and women [11]. Our study therefore seeks to disentangle the mechanisms and pathways through which work-related activities, socioeconomic status and stress impact on the health status of the elderly. More specifically, we aimed to examine whether stress defined in terms of time pressure plays a mediating role in the relationship between work-related activities and self-reported health.

## Methods

### Data

Data from the Multinational Time Use Study (MTUS, version W53) were used for this study. MTUS is a cross-national harmonized and comparative time-use database from 25 countries, collated and organized by the Centre for Time Use Research at the University of Oxford [43]. Diaries were self-administered followed by a personal visit in most countries. In the interview, diarist reported the total time spent on 41 activities over a 24-h period in 5, 10 or 15-min intervals during a randomly assigned day in a week in France, Italy and Spain, and two days (weekday and weekend) in the UK. In the Netherlands, diarist reported their time use activities for seven consecutive days [43]. For the purpose of this study, we limited our sample to participants who were 65 years and above and their time use activities sum up to 1440 min (24 h). The countries considered in the final analysis were the United Kingdom (survey year: 2000; *n* = 2870), Spain (survey year: 2002; *n* = 9889), Italy (survey year: 2002; *n* = 8709), France (survey year: 1998; *n* = 2231) and the Netherlands (survey year: 2000; *n* = 1764). These countries were selected based on the availability of the health variable in the respective diary collection.

### Measures and model specification

**Fig. 1:**
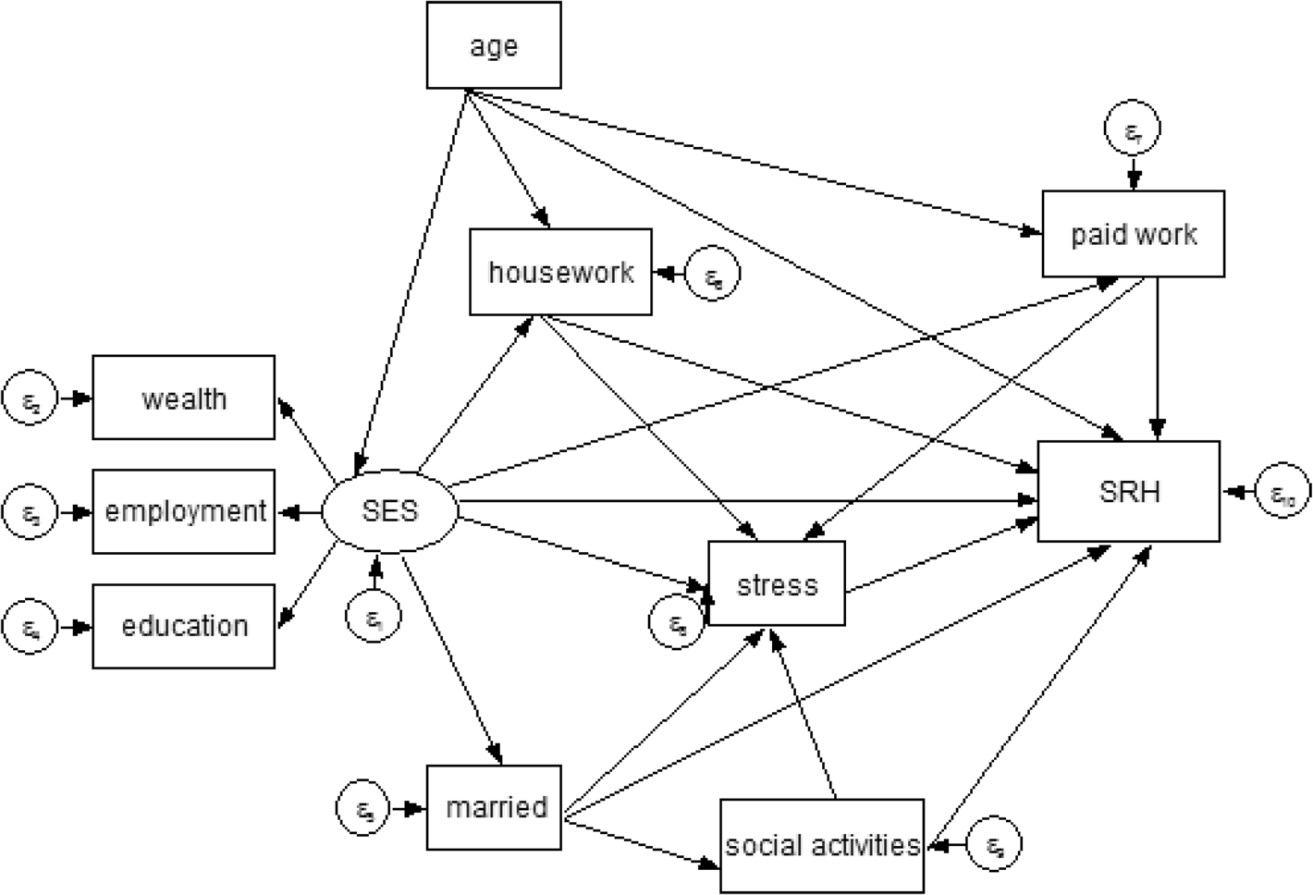
Conceptual model of self-reported Health (SRH), stress measured by time pressure, socioeconomic status (SES), demographic factors, social activities and work-related time use activities (housework and paid work) among the elderly

Structural equation models were used to test the proposed relationships between the concepts described in Fig. 1. This model reflected the hypothesized pathways between self-reported health and the psychosocial measures being assessed. Socioeconomic status (SES) was a latent variable, which was represented by a circle. This variable was measured by three observed indicators: education (less than secondary education, completed secondary education and above secondary education), wealth (measured by car ownership, and coded to indicate no car, one car and two or more cars) and employment status (not working for pay, currently in paid employment). Among older adults, these measures of SES have been shown to be associated with health [4, 44].

Self-reported health and stress in terms of time pressure, represented by rectangles, were the two key observed endogenous (dependent variables) used for this study. Self-reported health was assessed using the question: “How is your health in general; would you say that it is …? ” response options: zero (poor) to three (very good). We used the responses as a 4-level ordinal variable, where higher levels indicate better health. Intense time pressure, an indicator of stress [41], was measured using the following question: “Would you say you always feel rushed even to do the things you have to do, only sometimes feel rushed, or almost never feel rushed?” The responses were coded as: (1) never (2) sometimes and (3) always. It was used as an ordinal variable, with higher levels corresponding to stress.

In the model, we considered two work-related time use activities (housework and paid work), measured in hours per day, as these activities are associated with stress, depression and physical health status [26, 36, 45]. We also included marital status (married/cohabiting vs single/widowed) and age which previous studies have found to be associated with stress and general health status [46]. To investigate work-related time use activities and potential “stress buffers”, we used time devoted to social activities [41], (measured in hours per day) (Fig. 1). Additional file 1: Table S2 lists the detailed time use activities included in the 3 broad categories used for this study.

### Analytic strategy

The analytic strategy included four separate steps. First, descriptive analysis summarized gender differences in self-reported health, stress and other social factors including the mean time allocated to time use activities. Second, Pearson’s correlations coefficients (*r*) were estimated to examine the bivariate correlations of all measured variables. Third, a linear structural model was implemented and estimated using a maximum-likehood function [47]. This was aimed at estimating the direct and indirect relationship between self-reported health, stress, time use activities and other social factors. This can be expressed mathematically as follows:

1 $$ \mathrm{Y}=\mathrm{BY}+\Gamma X+\alpha +\varsigma $$

where, *Ү* = vector of the endogenous variables (self-reported health and stress); *X*= vector of the exogenous variables, both latent (SES) and observed (age, marital status, paidwork, housework and social activities); B and *Γ* = matrices of the coefficients; *α* = vector of the intercepts; *ς* = vector of the error terms.

Finally, a model for the indirect and total effects of housework and paidwork via stress was constructed. The assumption was that stress in terms of time pressure did not mediate the relationship between these work-related time use activities and self-reported health, due to time availability at old age [11]. We use the term “effect” in its technical sense and do not want to imply causation [48].

In order to test the hypothesis, we used stress as a mediating variable to estimate the indirect and total effects of these activities on self-reported health. The total effect was obtained through the summation of the direct and indirect effects using Stata’s *estat teffects* command, which can be expressed mathematically as follows:

2 $$ \mathrm{c}={\mathrm{c}}^{\prime }+\mathrm{ab} $$

Where, c = Total effect,  c^′^ = Direct effect,  ab = Indirect effect.

The chi-square (χ2) is the traditional measure for assessing the overall goodness of fit of an SEM model [49], however, because it is highly sensitive to large samples [49], we considered the Comparative Fit Index (CFI), and the Root Mean Square Error of Approximation (RMSEA) suggested by Hooper et al. [49] to evaluate model fit. An RMSEA less than 0.06 shows a good fit. The CFI, on the other hand, ranges between 0 and 1, where values closer to 1 indicate better model fit. A good fit was defined as values greater than 0.95 and values greater than 0.90 indicates an acceptable fit to the data [49]. The *estat mindices* command was used for the modification of the initial model and the final conceptual model was subsequently determined based on the chi-square (χ2) test of additional paths from a theoretical view point. The analyses were done separately for men and women. All statistical analyses were performed in STATA version 14 [50].

## Results

### Descriptive statistics

Table 1 provides the distribution information of respondents, stratified by gender and Additional file 1: Tables S3 and S4 by country and gender. We observed that more elderly women than elderly men reported poorer health (20.5% vs 15.1%). Women were more likely to report stress than men. Approximately 70.7% of elderly men reported never having any intense time pressure as compared to 58.3% of women. Gender differences were also found in socioeconomic factors. Women were on average older than men (73.1 years vs 72.4 years). However, more men than women were married or cohabiting (79.9% vs 47.1%) and they also had higher educational attainment as compared to women. About 11% of elderly men and 5% of women reported having a tertiary education (Table 1).

**Table 1:** General description of the study sample (in percentages, means, 95% CI and SD), men and women

	Men (*n* = 11,168)			Women (*n* = 14,295)		
	Mean/ %	SD	(95% CI)	Mean/ %	SD	(95% CI)
Self-reported health						
Poor	15.1		(14.4–15.8)	20.5		(19.9–21.1)
Fair	44.5		(43.6–45.4)	47.0		(46.1–47.8)
Good	32.5		(31.7–33.4)	26.1		(25.4–26.9)
Very good	7.9		(7.4–8.4)	6.4		(5.9–6.8)
Stress						
Almost never	70.7		(69.8–71.5)	58.3		(57.5–59.1)
Sometimes	23.3		(22.5–24.1)	31.7		(30.9–32.4)
Often	6.0		(5.5–6.4)	10.0		(9.4–10.4)
Sociodemographic & economic factors						
Age	72.40	5.01		73.06	5.13	
65–69	35.3		(34.3–36.1)	31.0		(30.2–31.7)
70–74	29.0		(28.1–29.7)	27.7		(26.9–28.4)
75–79	20.2		(19.4–20.9)	20.8		(20.1–21.5)
80+	15.6		(14.9–16.3)	20.5		(19.8–21.1)
Marital Status						
Single/widowed	20.2		(19.4–20.9)	52.9		(52.1–53.7)
Married/Cohabiting	79.9		(79.0–80.5)	47.1		(46.2–47.8)
Education						
Incomplete Sec. or less	60.9		(60.0–61.8)	73.3		(72.6–74.0)
Secondary completed	28.0		(27.1–28.8)	21.4		(20.7–22.1)
Tertiary Completed or above	11.1		(10.4–11.6)	5.2		(4.8–5.6)
Wealth						
Car ownership	2.46	1.45		1.84	1.61	
No car	24.1		(23.3–24.9)	41.6		(40.7–42.4)
1 car	52.5		(51.5–53.4)	39.4		(38.6–40.2)
2+ cars	23.4		(22.5–24.1)	19.0		(18.3–19.6)
Employment Status						
Not working for pay	94.9		(94.4–95.2)	98.1		(97.8–98.2)
Currently in paid employment	5.1		(4.7–5.5)	1.9		(1.7–2.1)
Time use Activities						
Paid work hours/day	0.26	1.36		0.08	0.71	
0 h	94.9		(94.5–95.3)	98.1		(97.8–98.3)
> 0 h	5.1		(4.6–5.4)	1.9		(1.6–2.1)
House work hours/day	2.82	2.51		4.85	2.63	
Less than 1 h	29.2		(28.3–30.0)	9.0		(8.5–9.4)
1 to 3 h	27.9		(27.0–28.7)	14.4		(13.8–14.9)
3 to 6 h	31.4		(30.4–32.2)	44.5		43.7–45.3)
> 6 h	11.6		(10.9–12.1)	32.1		(31.2–32.8)
Social activities hours/day	1.21	1.83		1.10	1.68	
Less than 2 h	76.2		(75.4–77.0)	79.0		(78.2–79.6)
2 to 4 h	16.6		(15.9–17.2)	15.2		(14.6–15.8)
> 4 h	7.2		(6.7–7.6)	5.8		(5.4–6.2)

Work-related time use activities (housework and paid work) varied considerably among elderly men and women. Women spent more time on housework activities (4.85 h/day vs 2.82 h/day), while men spent more time in paid work (0.26 h/day vs 0.08 h/day). However, a cross-country comparison in Additional file 1: Table S1 shows that the most time devoted to housework activities was found among women in Italy (5.15 h/day), while the least was observed in the Netherlands (4.44 h/day). Elderly women spent remarkably fewer hours in paid work compared to men. We observed that there were no differences in time devoted to paid work among women in Italy, Spain and France. The lowest value was observed in these countries (0.07 h/day), while most time spent in paid work was found in the Netherlands (0.11 h per day).

Regarding time allocation to social activities, men devoted on average 1.21 h/day to these activities as compared to 1.10 h/day for women. The highest value was found in the Netherlands for men and women (1.73 h/day vs 1.67 h/day), the least among men in Spain (0.97 h/day) and women in France (0.88 h/day).

### Bivariate analysis

The results of the bivariate analysis (Pearson correlation) between all measured variables are shown in Tables 2 (separated by gender).

**Table 2:** Correlations between all measured variables by gender

	(1)	(2)	(3)	(4)	(5)	(6)	(7)	(8)	(9)	(10)
Men										
Self-reported health (1)										
Stress (2)	−0.162***									
Age (3)	−0.165***	− 0.019*								
Married (4)	0.035***	0.026**	−0.153***							
Education (5)	0.201***	0.007	−0.081***	0.043***						
Employment status (6)	0.096***	0.095***	−0.120***	0.031***	0.087***					
Wealth (7)	0.135***	0.069***	−0.228***	0.120***	0.154***	0.138***				
Housework (8)	0.163***	0.022*	−0.129***	−0.052***	0.024**	−0.089***	0.062***			
Paid work (9)	0.082***	0.067***	−0.094***	0.017	0.081***	0.595***	0.114***	−0.125***		
Social support (10)	0.084***	−0.004	−0.099***	− 0.043***	0.062***	0.018	0.080***	−0.142***	− 0.042***	
Women										
Self-reported health (1)										
Stress (2)	−0.159***									
Age (3)	−0.169***	−0.086***								
Married (4)	0.054***	0.057***	−0.347***							
Education (5)	0.211***	−0.018*	−0.128***	0.058***						
Employment status (6)	0.091***	0.024***	−0.089***	0.024**	0.085***					
Wealth (7)	0.119***	0.054***	−0.187***	0.213***	0.125***	0.052***				
Housework (8)	0.165***	0.072***	−0.317***	0.240***	0.007	−0.036***	0.050***			
Paid work (9)	0.061***	0.021**	−0.075***	0.027*	0.089***	0.553***	0.053***	−0.037***		
Social support (10)	0.101***	−0.006	−0.057***	− 0.081***	0.045***	−0.011	0.014	−0.159***	− 0.029***	

*Notes*: *** *p* < 0.001; ** *p* < 0.01; * *p* < 0.05

Overall, the correlational pattern was very similar among women and men. The bivariate analysis showed that stress was negatively associated with self-reported health among elderly men (*r* = − 0.16) and women (*r* = − 0.17). All three measures of socioeconomic status including education, wealth and employment were positively associated with self-reported health among both genders. Educational attainment showed the strongest correlation among men (*r* = 0.20) and women (*r* = 0.21). Employment and wealth were positively associated with stress for both genders. Meanwhile, educational attainment was found to be negatively associated with stress among women (*r* = − 0.02), but not statistically significant for men. Age was significantly and negatively associated with self-reported and stress among elderly men and women. Housework and paid work were positively associated with self-reported health and stress. However, the correlation between stress and these time use activities were low. Surprisingly, social activities were not associated with stress for both genders.

### Estimates of direct, indirect and total associations

**Fig. 2:**
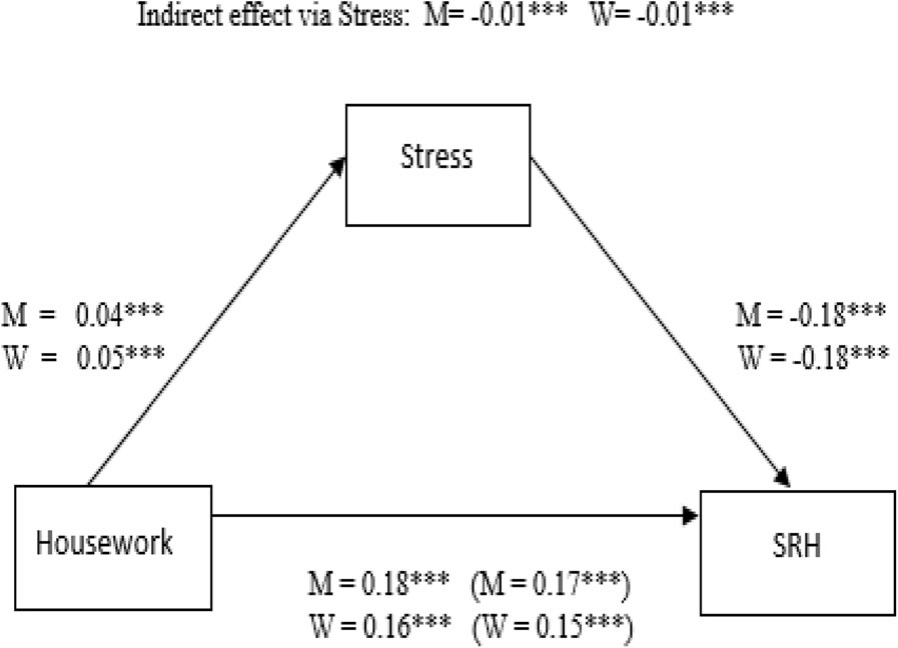
Indirect effect of housework on self-reported health (SRH) via stress. Standardized coefficients, adjusted for socioeconomic status (SES), age, marital status, social activities and work-related time use activities. Coefficients for the total effects in parentheses. M = men, W = women. *** *p* < 0.001; ** *p* < 0.01; * *p* < 0.05

**Fig. 3:**
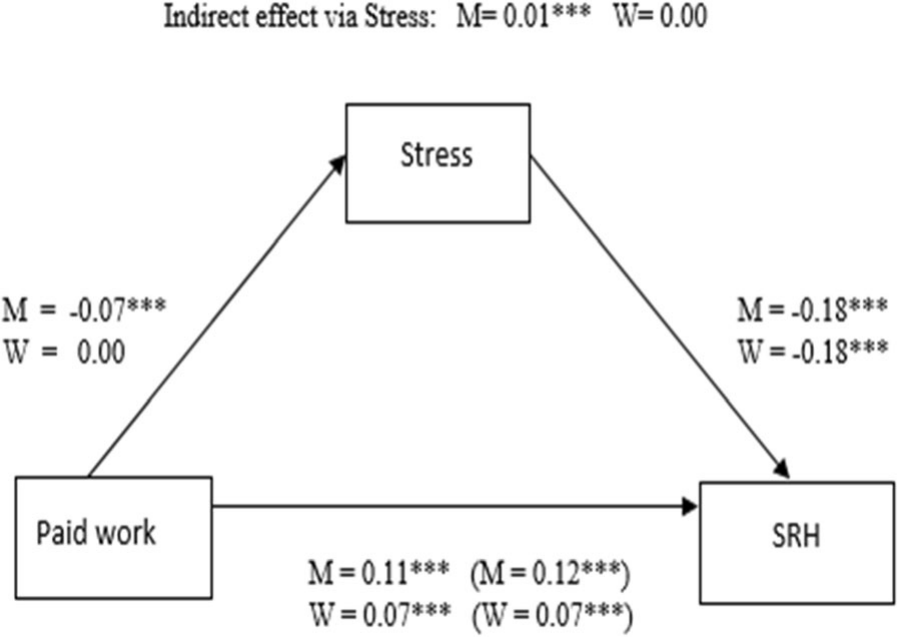
Indirect effect of paid work on self-reported health (SRH) via stress. Standardized coefficients, adjusted for socioeconomic status (SES), age, marital status, social activities and work-related time use activities. Coefficients for the total effects in parentheses. M = men, W = women. *** *p* < 0.001; ** *p* < 0.01; * *p* < 0.05

Table 3 and Figs. 2 and 3 present the estimated direct, indirect and total effects on key outcomes from the structural equation models.

**Table 3:** Standardized direct effects on key outcomes from the Structural Equation Model (SEM)

Path	Men	Women
Direct effects on Stress		
Age	0.003 (0.009)	− 0.060 (0.009)***
Married/Cohabitation	0.021 (0.010)**	0.024 (0.009)**
SES	0.182 (0.027)***	0.028 (0.021)
Housework	0.040 (0.009)***	0.049 (0.009)***
Paid work	−0.069 (0.025)**	− 0.003 (0.017)
Social activities	−0.001 (0.009)	0.001 (0.009)
Direct effects on Self-reported health		
Age	−0.118 (0.009)***	−0.125 (0.008)***
Married/Cohabitation	0.033 (0.009)***	−0.009 (0.008)
SES	0.237 (0.036)***	0.328 (0.054)***
Stress	−0.176 (0.009)***	−0.181 (0.007)***
Housework	0.182 (0.009)***	0.162 (0.008)***
Paid work	0.108 (0.009)***	0.065 (0.008)***
Social activities	0.103 (0.009)***	0.119 (0.008)***

*Notes*: Significance level: *** *p* < 0.001; ** *p* < 0.01; * *p* < 0.05. Observed Information Matrix (OIM) standard errors in parentheses. Model fit: (CFI = 0.92; RMSEA = 0.056, with 90% C.I. = 0.054–0.059), and women (CFI = 0.89; RMSEA = 0.064, with 90% C.I. = 0.062–0.068)

Overall, SES had the greatest direct positive effect on stress among men (*β* = 0.182). Among women, housework had the greatest positive effect on stress (*β* = 0.049), followed by SES (*β* = 0.028). Meanwhile, paid work had a negative effect on stress among men (*β* = − 0.069), but it was not statistically significant among women. Nonetheless, both paid work and housework had a positive effect on self-reported health among older men and women. Although stress had a negative direct influence on self-reported health among men and women, we found that the paths from paid work and housework on self-reported health via stress (mediator) were very weak because the indirect effects were close to zero among both genders (Figs. 2 and 3). Hence, there was almost no difference between the direct and total effects of these work-related time use activities on self-reported health among elderly men and women.

## Discussion

The primary objective of the current study was to investigate the complexity of the relationships between work-related time use activities (housework and paid work), socioeconomic status, stress in terms of time pressure and self-reported health among older people in high-income countries with particular attention to gender differences in the effects. Our study not only analyzed the direct effects but also indirect and total effects, in order to disentangle the mechanisms and pathways through which these variables impact the health status of elderly men and women. The underlying premise of the study was that psychosocial factors influence the health status of older adults, however, these factors may also have an indirect differential impact among elderly men and women. Stress defined in terms of time pressure was thus viewed as a potential mediator in the relationships between social factors and health status (Fig. 1). Our study showed significant direct and indirect relationships between psychosocial factors and self-reported health among men and men, but there were gender differences in the magnitude of the associations. The key findings of the study can be summarized as follows. First, time devoted to housework and paid work was positively associated with self-reported health among elderly men and women. Second, whereas housework was positively associated with stress in both genders, paid work was negatively associated with stress only among men. Third, high levels of stress had a direct negative impact on self-reported health among elderly men and women, however, we confirmed that stress in terms of time pressure does not play a mediating role in the relationship between housework, paid work and self-reported health. Finally, social activities had a positive impact on self-reported health but had no significant impact on stress among older men and women.

### Relationship of the socioeconomic status, time use activities with the health outcomes by gender

Prior evidence suggests that people with low SES are more likely to report poorer health compared to those with higher SES [18, 51, 52]. However, there may be variations in gender differences in vulnerability to socioeconomic status on health outcomes [53, 54], where women are more likely than men to report poorer physical health [11] and psychological distress [36]. In our study, we found that SES measured by education, wealth and employment was an independent predictor of health outcomes among elderly men and women. In line with previous studies [55, 56], we found a positive relationship between higher SES and good health status among the older population. A possible explanation for this outcome is that individuals with low SES may lack access to physical, psychological and environmental resources [52]. Also, it has been hypothesized that low SES leads to greater exposure to stress [18]. In contrast to this hypothesis, our direct effect models showed that stress in terms of time pressure is higher as SES increases among older men, but not statistically significant for women. The explanation to the diverging findings may be due to the measurement concept of stress [41], as this study only considered intense time pressure and not stressful life events such as financial strain [18]. Meanwhile, a recent study by Talala et al. [57] on SES and the distribution of stress found a reverse and curvilinear relationships.

The growing literature documenting partnership status and health among the elderly suggest that married [58, 59] and cohabiting older adults have better health than their unpartnered counterparts [60]. Although a variety of explanations have been given for these differences [61], social isolation and depression among single individuals are some of the psychological factors attributed to this physical health outcome [62]. However, data from several studies suggest that the protective effects of marriage on health are unequal among older men and women [63, 64] and that marriage appears to be more beneficial to men’s health compared to women [64–66]. Results from the current study showed a positive relationship between marriage and self-reported health only among men. As previously noted [37], a possible explanation for this phenomenon may be gender differentials in stress exposure from marital responsibilities. Nevertheless, the direct effects of relationship status in the present study indicated that both marriage/cohabitation was positively associated with stress as measured by time pressure among both men and women. This evidence and that of earlier studies [67] suggest that although marriage has an overall health benefit, it may not be a ‘buffer’ of stress even among the elderly. This is because the key sources of stress associated with marital roles [68] and poor marital quality among young and middle-aged adults [69] may be present among the older population.

Age was also significantly correlated with health in both elderly men and women, in line with the claim that the prevalence of good physical health decreases as age increases [11, 70]. However, age was negatively associated with stress among women but not statistically significant for men, a finding consistent with previous research that showed that older people experience less daily stress compared to midlife and younger adults [71, 72]. This is mainly due to the differential roles of the elderly compared to younger adults. For instance, children are key sources of daily stress for working-age adults and older adults usually do not have the same parental roles and responsibilities with respect to child-rearing [71].

Time devoted to social activities was positively associated with health status for both older men and women, consistent with prior studies [73–75]. Nevertheless, time allocation to social activities varied among elderly men and women across countries. In general, men allocated more time to social activities than women in all countries, except for Spain (Additional file 1: Table S1). Previous studies have also stressed the importance of older adult’s participation in social support and network activities such as religious activities [74], social or other clubs [76] for psychological well-being [77] and increase in survival among older people [78]. For example, a study by Engelhardt et al. [79] found that social involvement enhances cognitive functioning among the elderly. Also, social participation has been found to be related to low level of stress and depression among the elderly [77, 80]. We therefore expected social activities to ameliorate stress among older adults, however, we found no significant direct relationship between social support activities and stress among elderly men and women. One possible reason for the lack of association may be related to measurement issues. Social activities was measured by the amount of time spent on activities such as religious activities, visiting friends, excursions and observer sports (Additional file 1: Table S2), and not measuring the quality of social support and network size [81]. These aspects of social network have been found as protective factors against stress in prior research [82].

Work-related time use activities (i.e. housework and paid work) were directly associated with both self-reported health and stress. Paid work was negatively associated with stress among men, but this association was not found among women. Although the amount of time allocated to paid work activities among the elderly was very small compared to young adults [11], we found paid work at older ages to be directly linked with reduced stress among men. This is consistent with previous findings that support the reduced role-strain hypothesis [83], that suggests that older adults may engage in less demanding and part-time jobs after retirement, which might be less stressful for them, especially for retired men [83]. Even though we were unable to account separately for older adults’ engagement in part-time work in our sample, we found that only about 5% and 2% of elderly men and women respectively in Western Europe were in paid employment (Table 1). Recent figures also shows that less than 5% of elderly people in Europe aged 65 years and above were still employed. Nevertheless, the employment rate for the subgroup 65–69 years has increased from 8.8% in 2005 to 10.5% in 2011 [84]. In the US, a higher participation rate in paid employment after retirement than in Europe has been noted [11, 84]. The explanation is that most Western European countries have universal social and healthcare systems [84]. In contrast, the high cost of health care in the US may account for the high employment rate among the older population [85]. Currently, it is still unclear whether working at old age is beneficial to older adult’s physical health [11, 38, 86]. Nonetheless, this current study found paid work at old age to be positively related with self-reported health for both genders, as found in some previous studies [11, 86], but the magnitude of the effect varied among men and women [86]. Perhaps, the social network that older adults gain or maintain at their workplace [86] combined with low levels of depressions at old age, due to the reduction in the amount of time devoted to paid work activities [83] may explain these favorable direct correlations between paid work and self-reported health among the elderly.

Regarding time devoted to housework, the result showed gender and cross-national variations (Additional file 1: Table S1). Furthermore, consistent with previous evidence [11, 26, 29, 31, 32, 39], we found that women devote more to housework activities than men. In spite of these gender differences, time devoted to housework was directly and positively related with self-reported health among both genders, consistent with prior evidence [11, 39], although the investigations of the association between time devoted to housework and health in prior research were inconsistent [11, 39, 87, 88]. For example, Lawlor et al. [88] found no association between heavy housework activities and reduced levels of being overweight among older British women. Similarly, a study conducted in China by Wen et al. [87] found negative associations between health status and various types of housework activities among women. On the other hand, Adjei and Brand [39] suggested that some hours devoted to housework activities might improve the health of the elderly. This inconsistency may be due to the different contexts and health outcomes [11, 87].

While we did not find any direct negative impact of housework on self-reported health in this current study, we did find this when examining stress in terms of time pressure among both genders. When potential indirect pathways were examined, mediation analysis did not show an indirect effect of housework on self-reported health via stress. Among the working population, prior studies showed that working-age adults who devote more time to housework activities experience high levels of stress and increased depression [36, 37]. It has been suggested that multiple role demands and work overload may be possible explanations for this psychological health outcome [41, 89]. However, since it was suggested in previous studies [11, 39] that the positive relationship between work-related time use activities and physical health is attributable to less stress at old age, we therefore expected to find support for the time availability theory suggesting that older adults after retirement may not have the same time pressure as younger adults. Indeed, in line with these expectations, we found that stress in terms of time pressure does not mediate or indirectly influence the positive associations between housework, paid work and self-reported health among elderly men and women.

Our analysis is not without limitations. First, the cross-sectional design of the research prevents conclusions about causality, and it is not possible to determine directionality in the relationship between the investigated factors and self-reported health. Second, although we have controlled for a variety of confounders, biological and behavioral determinants of health status among older adults [12, 15] were not included in the theoretical model, due to data constraints. Third, this study used subjective rather than objective reports of time use activities and health status. However, estimates from dedicated time use surveys are more reliable and accurate than survey estimates [90, 91]. Furthermore, self-reported health has consistently been shown to be a valid measure of current health status [92]. Nonetheless, we acknowledge that there may be gender differences in health reporting behavior [93]. Fourth, due to data limitations, this study relied on time use surveys that have been collected at different points in time with variations in the modes of data collection in the chosen countries, however, evaluation studies suggest that these differences do not affect the comparability of the data [94]. Notwithstanding these limitations, the study provided the first overview of the inter-related pathways through which psychosocial factors impact the health of older adults using a large-scale and homogeneous time use data set from Europe.

## Conclusions

The results from the SEM models provided evidence of the interrelating paths between psychosocial factors and health status among elderly men and women in western European countries. Our findings suggest that although stress in terms of time pressure has a strong direct negative effect on health, it does not indirectly influence the positive effects of work-related time use activities on self-reported health among elderly men and women. The results support the time availability hypothesis that older adults may not have the same time pressure as younger adults after retirement.

## Additional file

Additional file 1: **Table S1.** General description of time use activities (means and SD), men and women, by country. **Table S2.** Typology of activities. **Table S3.** General description of the study sample (in percentages) by country, men. **Table S4.** General description of the study sample (in percentages) by country, women. (DOCX 35 kb)

## Abbreviations

CFI: Comparative Fit Index
MTUS: Multinational Time Use Study
RMSEA: Root Mean Square Error of Approximation
SEM: Structural Equation Modelling
SES: Socioeconomic Status
