# Supplementary material for: Time spent on work-related activities, social activities and time pressure as intermediary determinants of health disparities among elderly women and men in 5 European countries: a structural equation model
Source: Int J Equity Health. 2018 Aug 16;17:121. doi: 10.1186/s12939-018-0840-y (PMC6097401; doi:10.1186/s12939-018-0840-y)
Supplement: Supplementary file 1 — Table S1. General description of time use activities (means and SD), men and women, by country. Table S2. Typology of activities. Table S3. General description of the study sample (in percentages) by country, men. Table S4. General description of the study sample (in percentages) by country, women. (DOCX 35 kb) [file 12939_2018_840_MOESM1_ESM.docx]

**Supporting Information**

**S1 Table**. General description of time use activities (means and SD), men and women, by country

|  |  |  |  |  |  |  |
| --- | --- | --- | --- | --- | --- | --- |
|  | Housework hours/day |  | Paidwork hours/day |  | Social support hours/day |  |
|  | Mean | SD | Mean | SD | Mean | SD |
| **Men** |  |  |  |  |  |  |
| Italy | **2.69** | 2.48 | **0.38** | 1.68 | **1.38** | 1.77 |
| Spain | **2.44** | 2.51 | **0.22** | 1.22 | **0.97** | 1.59 |
| France | **3.02** | 2.18 | **0.17** | 1.14 | **1.03** | 1.63 |
| Netherlands | **3.69** | 2.64 | **0.18** | 1.03 | **1.73** | 2.60 |
| UK | **3.84** | 2.36 | **0.20** | 1.09 | **1.37** | 2.13 |
| **Women** |  |  |  |  |  |  |
| Italy | **5.14** | 2.74 | **0.07** | 0.72 | **1.20** | 1.64 |
| Spain | **4.77** | 2.71 | **0.07** | 0.67 | **1.20** | 1.43 |
| France | **4.73** | 2.28 | **0.07** | 0.65 | **0.88** | 1.51 |
| Netherlands | **4.44** | 2.36 | **0.11** | 0.82 | **1.67** | 2.43 |
| UK | **4.55** | 2.24 | **0.09** | 0.79 | **1.23** | 1.96 |

| **S2 Table**. Typology of activities | | |  |  |  |  |
| --- | --- | --- | --- | --- | --- | --- |
| **Broad categories of activity** | | | **Name of variable** **(harmonised)** | | **Description** | |
| **1. Paid work** | | | AV01 | | Paid work | |
|  | | | Av02 | | Paid work at home | |
|  |  |  | AV03 | | Second job | |
|  |  |  | AV05 | | Travel to/ from work | |
|  |  |  |  |  |  |  |
| **2.Housework** | | | AV06 | | Cooking/Washing up | |
|  |  |  | AV07 | | Housework | |
|  |  |  | AV08 | | Odd jobs | |
|  |  |  | AV09 | | Gardening, pets | |
|  |  |  | AV10 | | Shopping | |
|  |  |  | AV12 | | Domestic travel | |
|  |  |  |  |  |  |  |
| **3.Social support** |  |  | AV17 | | Leisure travel | |
|  |  |  | AV18 | | Excursions | |
|  |  |  | AV22 | | Religious activities | |
|  |  |  | AV24 | | Cinema, theatre | |
|  |  |  | AV26 | | Social club | |
|  |  |  | AV27 | | Pub | |
|  |  |  | AV28 | | Restaurant | |
|  |  |  | AV29 | | Visiting friends | |
|  |  |  | AV04 | | School/classes | |
|  |  |  | AV20 | | Passive/observer sports | |
|  |  |  |  |  |  |  |

**S3 Table.** General description of the study sample (in percentages) by country, men

|  | **France** | **Italy** | **Netherlands** | **Spain** | **UK** |
| --- | --- | --- | --- | --- | --- |
| **Variables** | (*n*=1,118) | (*n*=3,770) | (*n*=812) | (*n*=4,231) | (*n*=1,237) |
| ***Self-reported health*** |  |  |  |  |  |
| Poor | 10.4 | 11.9 | 2.6 | 22.6 | 11.7 |
| Fair | 42.5 | 61.5 | 26.7 | 35.9 | 35.3 |
| Good | 39.1 | 23.8 | 56.0 | 34.3 | 31.7 |
| Very good | 8.1 | 2.8 | 14.7 | 7.2 | 21.3 |
| ***Stress*** |  |  |  |  |  |
| Almost never | 69.4 | 55.5 | 82.8 | 82.9 | 68.5 |
| Sometimes | 24.3 | 35.2 | 13.8 | 13.2 | 27.4 |
| Often | 6.3 | 9.3 | 3.5 | 3.9 | 4.1 |
| ***Sociodemographic & economic factors*** |  |  |  |  |  |
| *Age* |  |  |  |  |  |
| 65-69 | 34.4 | 35.0 | 40.5 | 34.7 | 35.5 |
| 70-74 | 32.0 | 29.2 | 31.0 | 27.8 | 28.0 |
| 75-79 | 23.5 | 19.6 | 19.8 | 19.6 | 21.1 |
| 80+ | 10.1 | 16.1 | 8.6 | 18.0 | 15.4 |
| *Civic Status* |  |  |  |  |  |
| Not married | 16.8 | 19.1 | 21.6 | 20.1 | 25.8 |
| Married/Cohabiting | 83.2 | 80.9 | 78.5 | 79.9 | 74.2 |
| *Education* |  |  |  |  |  |
| Incomplete Sec. or less | 25.9 | 67.5 | 35.3 | 68.3 | 64.4 |
| Secondary completed | 52.5 | 27.7 | 36.2 | 23.2 | 17.7 |
| Tertiary Completed or above | 21.7 | 4.8 | 28.5 | 8.5 | 18.0 |
| *Wealth* |  |  |  |  |  |
| Car ownership |  |  |  |  |  |
| No car | 0.0 | 16.0 | 1.7 | 40.9 | 27.9 |
| 1 car | 78.0 | 48.4 | 83.6 | 42.0 | 57.6 |
| 2+ cars | 22.0 | 35.6 | 14.7 | 17.1 | 14.6 |
| *Employment Status* | |  |  |  |  |
| Not working for pay | 98.7 | 92.4 | 97.4 | 96.3 | 92.4 |
| Currently in paid employment | 1.3 | 7.6 | 2.6 | 3.7 | 7.6 |
| ***Time use Activities*** |  |  |  |  |  |
| Paid work hours/day |  |  |  |  |  |
| 0 hours | 96.2 | 94.2 | 95.3 | 95.0 | 95.3 |
| >0 hours | 3.8 | 5.8 | 4.7 | 5.0 | 4.7 |
| House work hours/day |  |  |  |  |  |
| Less than 1 hours | 18.7 | 31.0 | 18.0 | 37.7 | 11.8 |
| 1 to 3 hours | 33.6 | 28.7 | 25.5 | 26.8 | 25.9 |
| 3 to 6 hours | 38.2 | 29.9 | 40.0 | 25.6 | 43.4 |
| >6 hours | 9.5 | 10.5 | 16.5 | 9.9 | 18.9 |
| Social support hours/day |  |  |  |  |  |
| Less than 2 hours | 80.5 | 71.3 | 68.1 | 81.5 | 74.6 |
| 2 to 4 hours | 13.2 | 20.9 | 17.7 | 13.7 | 15.6 |
| >4 hours | 6.3 | 7.8 | 14.2 | 4.8 | 9.8 |

**S4 Table.** General description of the study sample (in percentages) by country, women

|  | **France** | **Italy** | **Netherlands** | **Spain** | **UK** |
| --- | --- | --- | --- | --- | --- |
| **Variables** | (*n*=1,113) | (*n*=4,939) | (*n*=952) | (*n*=5,658) | (*n*=1,633) |
| ***Self-reported health*** |  |  |  |  |  |
| Poor | 12.9 | 18.8 | 2.9 | 29.0 | 11.8 |
| Fair | 45.6 | 64.3 | 31.6 | 38.4 | 34.5 |
| Good | 36.4 | 15.2 | 49.3 | 27.5 | 34.2 |
| Very good | 5.1 | 1.7 | 16.2 | 5.2 | 19.5 |
| ***Stress*** |  |  |  |  |  |
| Almost never | 57.1 | 46.5 | 64.7 | 68.2 | 56.9 |
| Sometimes | 36.2 | 39.5 | 30.9 | 22.4 | 37.8 |
| Often | 6.7 | 14.0 | 4.4 | 9.4 | 5.3 |
| ***Sociodemographic & economic factors*** |  |  |  |  |  |
| *Age* |  |  |  |  |  |
| 65-69 | 38.5 | 29.5 | 33.8 | 30.4 | 30.8 |
| 70-74 | 31.1 | 26.9 | 30.2 | 27.2 | 28.3 |
| 75-79 | 20.5 | 20.7 | 23.5 | 19.8 | 23.2 |
| 80+ | 10.0 | 22.9 | 12.5 | 22.6 | 17.7 |
| *Civic Status* |  |  |  |  |  |
| Not married | 30.9 | 57.3 | 60.3 | 51.5 | 55.2 |
| Married/Cohabiting | 69.1 | 42.7 | 39.7 | 48.5 | 44.8 |
| *Education* |  |  |  |  |  |
| Incomplete Sec. or less | 28.7 | 80.1 | 59.6 | 77.7 | 76.5 |
| Secondary completed | 55.0 | 17.9 | 31.6 | 18.5 | 13.7 |
| Tertiary Completed or above | 16.4 | 2.1 | 8.8 | 3.9 | 9.8 |
| *Wealth* |  |  |  |  |  |
| Car ownership |  |  |  |  |  |
| No car | 0.0 | 38.3 | 13.2 | 55.4 | 48.7 |
| 1 car | 81.6 | 34.1 | 53.7 | 32.1 | 43.8 |
| 2+ cars | 18.4 | 27.6 | 33.1 | 12.5 | 7.5 |
| *Employment Status* | |  |  |  |  |
| Not working for pay | 98.5 | 98.4 | 97.8 | 98.4 | 95.7 |
| Currently in paid employment | 1.5 | 1.6 | 2.2 | 1.6 | 4.4 |
| ***Time use Activities*** |  |  |  |  |  |
| Paid work hours/day |  |  |  |  |  |
| 0 hours | 97.6 | 98.8 | 96.7 | 97.8 | 98.0 |
| >0 hours | 2.4 | 1.2 | 3.3 | 2.2 | 2.0 |
| House work hours/day |  |  |  |  |  |
| Less than 1 hours | 5.4 | 9.4 | 6.3 | 10.8 | 5.7 |
| 1 to 3 hours | 15.0 | 12.0 | 20.4 | 14.2 | 18.8 |
| 3 to 6 hours | 53.6 | 40.6 | 49.9 | 43.4 | 51.0 |
| >6 hours | 26.0 | 38.0 | 23.4 | 31.7 | 24.5 |
| Social support hours/day |  |  |  |  |  |
| Less than 2 hours | 83.2 | 74.6 | 68.3 | 84.2 | 77.2 |
| 2 to 4 hours | 11.9 | 19.8 | 17.4 | 11.8 | 14.3 |
| >4 hours | 4.9 | 5.6 | 14.3 | 4.0 | 8.6 |
